# Neuronal Antibodies in Children with or without Narcolepsy following H1N1-AS03 Vaccination

**DOI:** 10.1371/journal.pone.0129555

**Published:** 2015-06-19

**Authors:** Simon Thebault, Patrick Waters, Matthew D. Snape, Dominic Cottrell, Niklas Darin, Tove Hallböök, Anne Huutoniemi, Markku Partinen, Andrew J. Pollard, Angela Vincent

**Affiliations:** 1 Neuroimmunology Group, Nuffield Department of Clinical Neurosciences, Oxford University, Oxford, United Kingdom; 2 Department of Paediatrics, John Radcliffe Hospital, University of Oxford, Oxford, United Kingdom; 3 The NIHR Oxford Biomedical Centre, Oxford University Hospitals Trust, Oxford, United Kingdom; 4 Imperial College of Medicine, University of London, London, United Kingdom; 5 Department of Paediatrics, Institute of Clinical Sciences, Sahlgrenska Academy, University of Gothenburg, Gothenburg, Sweden; 6 Helsinki Sleep Clinic, Finnish Narcolepsy Research Centre, Helsinki, Finland; 7 Department of Clinical Neurosciences, University of Helsinki, Helsinki, Finland; Oasi Research Institute, ITALY

## Abstract

Type 1 narcolepsy is caused by deficiency of hypothalamic orexin/hypocretin. An autoimmune basis is suspected, but no specific antibodies, either causative or as biomarkers, have been identified. However, the AS03 adjuvanted split virion H1N1 (H1N1-AS03) vaccine, created to protect against the 2009 Pandemic, has been implicated as a trigger of narcolepsy particularly in children. Sera and CSFs from 13 H1N1-AS03-vaccinated patients (12 children, 1 young adult) with type 1 narcolepsy were tested for autoantibodies to known neuronal antigens including the N-methyl-D-aspartate receptor (NMDAR) and contactin-associated protein 2 (CASPR2), both associated with encephalopathies that include disordered sleep, to rodent brain tissue including the lateral hypothalamus, and to live hippocampal neurons in culture. When sufficient sample was available, CSF levels of melanin-concentrating hormone (MCH) were measured. Sera from 44 H1N1-ASO3-vaccinated children without narcolepsy were also examined. None of these patients’ CSFs or sera was positive for NMDAR or CASPR2 antibodies or binding to neurons; 4/13 sera bound to orexin-neurons in rat brain tissue, but also to other neurons. MCH levels were a marginally raised (n = 8; p = 0.054) in orexin-deficient narcolepsy patients compared with orexin-normal children (n = 6). In the 44 H1N1-AS03-vaccinated healthy children, there was no rise in total IgG levels or in CASPR2 or NMDAR antibodies three weeks following vaccination. In conclusion, there were no narcolepsy-specific autoantibodies identified in type 1 narcolepsy sera or CSFs, and no evidence for a general increase in immune reactivity following H1N1-AS03 vaccination in the healthy children. Antibodies to other neuronal specific membrane targets, with their potential for directing use of immunotherapies, are still an important goal for future research.

## Introduction

Narcolepsy is a lifelong and disabling condition, first described over 130 years ago [1]. It is characterised by dysregulation of the sleep-wake cycle with inappropriate penetration of rapid eye movement (REM) sleep, and cataplexy, a sudden loss of motor tone triggered by emotion. Type 1 narcolepsy[2] is associated with a selective loss of neurons secreting neuropeptides orexin A and B, also called hypocretins 1 and 2 [3]. The disease is diagnosed from the history of severe sleepiness, in addition to the co-existence of cataplexy, and a positive multiple sleep latency test (MSLT), or very low or absent CSF orexin [4].

A tightly-linked HLA association is well established [5]. Twin discordance,[6] and the association of onset with streptococcal infection [7] and pandemic flu immunisation [8] suggest an immunological trigger. In addition to the DQB1*06:02 association, [9] genome-wide association studies found polymorphisms in the T cell receptor alpha [10] as well as other immunity-linked genes. Antibodies to various CNS proteins, or candidate antigens, have been identified in narcolepsy using a variety of approaches, but none have yet led to development of disease-specific antibody tests (reviewed in [11–13]), and immunotherapies have produced variable results (reviewed in [13]).

Sleep disturbance is a common features of autoimmune forms of encephalitis associated with antibodies to the voltage-gated potassium channel (VGKC) complex proteins, contactin-associated protein 2 (CASPR2), or leucine- rich glioma-inactivated 1 (LGI1), or to N-methyl-D-aspartate receptor (NMDAR) which are all proteins exposed on the surface of live neurons (reviewed in [14]).

The recent recognition of children with narcolepsy and cataplexy, both presenting within months of Pandemrix vaccination (AS-03 adjuvanted) against the Pandemic 2009 H1N1 Swine Flu (H1N1-AS03), especially in northern European countries, [8, 15–18], provided an opportunity to look for potentially pathogenic antibodies in sera and CSFs close to onset. In addition, sera from healthy children before and after H1N1-AS03 vaccination [19] were examined for changes in total IgG and for CASPR2 and NMDAR antibodies.

## Methods

### Ethics statement

The sera and CSFs from narcolepsy patients were obtained with ethical permission and written informed consent from subjects or their parents with approval of the coordinating Ethical Boards of the University Hospital of Gothenburg and the Hospital District of Helsinki and Uusimaa. The study of H1N1 vaccination sera [19] was approved by the Oxfordshire Research Ethics Committee A (No 09/H0604/107), and the UK Medicines and Healthcare Products Regulatory Agency (EUDRACT 2009-014719-11).

All animal procedures were carried out in accordance with the UK Home Office guidelines under a project license granted by the Home Office to AV (Immunity in Neurological and Developmental Disorders, PPL No 30/1890). The adult rats were PBS-perfused under terminal barbiturate anaesthesia, and the newborn pups sacrificed by decapitation (Schedule 1). Compliance to rules and regulations and adherence to the 3Rs principles was monitored by Biomedical Services, University of Oxford and Home Office inspectors.

### Post H1N1-AS03 vaccination narcolepsy samples

The initial cohort was composed of 14 serum/CSF pairs from Swedish and Finnish patients from whom the initial association of narcolepsy after vaccination with Pandemrix were identified (Table 1) as described in detail elsewhere [17]. All were DQB1*06:02 positive. Sera from 15 children, including five who had had H1N1 vaccination but had normal hypocretin/orexin levels were used as controls. CSF orexin levels were measured using a commercially available radioimmunoassay (Phoenix Peptides, Arizona, USA). MCH levels were also measured by radioimmunoassay (Phoenix Peptides, Arizona, USA), but to ensure levels were within the linear range, 40 pg of the MCH standard was added before testing to each 100 μl sample (to give a final concentration of >400 pg/ml). Results were read from the standard curve (calculated by Graphpad Prism 5), and presented after subtraction of 40 pg for each sample.

**Table 1:** Narcolepsy patient demographics and results of antibodies to neuronal antigens.

Patient	Sex, age (y), time since onset (m)	CSF orexin A, MCH (pg/ml)	NMDAR (1:20)	CASPR2 (1:100)	LGI1 (1:20)	Contactin2 (1:100)	Dopamine D2 (1:20)	GABA_A3_ (1:20)	Immuno-fluorescence Tissue sections	Immuno-fluorescence Live hippocampal neurons
1	F,16, 13	<10, n.d.	0	0	0	0	0	0	0	0
2	F,15, 13	<10, 133	0	0	0	0	0	0	0	0
3	M, 4,16	<10, n.d.	0	0	0	0	0	0	0	0
4	M, 9, 10	52, n.d.	0	0	0	0	0	0	0	0
5	F, 8, 7	<10, n.d.	0	0	0	0	0	0	0	0
6	M, 11, 14	<10, 141	0	0	0	0	0	0	0	0
7	M, 12, 12	<10, 263	0	0	0	0	0	0	0	0
8	M, 16, 7	<10, 217	0	0	0	0	0	0	2	0
9	M, 14, 16	<10, n.d.	0	0	0	0	0	0	2	0
10	M, 18, 13	<10, 246	0	0	0	0	0	0	0	0
11*	M, 24, 41	<10, 198	0	0	0	0	0	0	0	0
12	F, 18, 20	<10, n.d.	0	1	0	0	0	0	2	0
13	M, 17, 19	55, 298	0	0	0	0	0	0	1	0
All narcolepsy patients (n = 13)		Orexin <110 pg/ml (n = 13), MCH 213+/- 57 pg/ml (n = 8)	0/13	1/13	0/13	0/13	0/13	0/13	4/13	0/13
Orexin normal paediatric sera/CSFs (n = 15)		Orexin >200 pg/ml (n = 15), MCH 156+/- 38 pg/ml (n = 6)	0/15	0/15	0/15	0/15	0/15	0/15	0/15	0/15

CSF orexin levels <110 pg/ml supports a diagnosis of type 1 narcolepsy (narcolepsy with cataplexy). MCH levels were measured in CSFs the CSFs that were still available and in one additional narcolepsy CSF (without a paired serum). Serum cell-based assays for antibodies to individual antigens, immunofluorescence for binding to orexin neurons on rodent brain tissue sections or to hippocampal neuronal cultures were scored subjectively by comparison with positive and negative controls: 0 = no binding, 1 = weakly positive, 2 = moderately positive, 3 = strongly positive, 4 = complete co-localisation.

*In patient 11 the onset of narcolepsy was 18 months before Pandemrix vaccination but his cataplexy worsened after vaccination. In all other cases the onset of symptoms was after Pandemrix vaccination.

### Pre and post vaccination healthy paediatric samples

Sera were obtained from 42 participants before and 3 weeks after 2 doses of an AS03-H1N1 adjuvanted split-virion vaccine (Pandemrix, GlaxoSmithKline, Rixenstart, Belgium). These samples had originally been obtained to investigate H1N1 seroconversion rates following vaccination [19].

### Serum IgG concentration

Serum IgG was measured by western blot using 10 μl of a 1:300 dilution of serum and detecting with peroxidase-labelled anti-human IgG. The blots were scanned and densitometric analysis performed of the 50 kDa IgG heavy chain; IgG levels were calculated by comparison with a human IgG heavy chain standard curve.

### Antibody assays to detect specific neuronal surface antibodies

Cell-based assays (eg.[20]) were used to measure antibodies to the VGKC-complex components, contactin2, CASPR2 (sera diluted 1:100) and LGI1, NMDAR, GABA_A3_ and dopamine (D2R) receptors (sera diluted 1:20). CSFs were tested at 1:1. The test samples were coded and mixed randomly with other samples for all tests. Binding of IgG antibodies to the surface of the antigen-expressing cells was detected with Alexa Fluor 568 (red) anti-human IgG (Invitrogen, UK). Results were scored by ST and one independent observer, in a double-blinded manner, according to a subjective but reproducible scale (0 = none, 1 = weak, 2 = moderate, 3 = strong, 4 = very strong; see [20]) and by comparison with positive and negative controls.

### Immunohistochemistry on rat brain frozen sections

Coronal hypothalamic 12 μm sections were made from snap-frozen brains of adult rats. They were subsequently, dried, fixed in 3% formaldehyde (1 minute, room temperature), and blocked in 10% foetal calf serum (1 hour, room temperature). The orexin neurons were first localised with anti-orexin-A antibody (Phoenix Pharmaceuticals, California, USA 1:150, overnight at 4°C). To detect binding of autoantibodies, sera (1:300) or CSFs (1:1) were applied overnight at 4°C, followed by Alexa Fluor 488 anti-human IgG (Invitrogen; 1 hour, room temperature). All steps were separated by three 1 min washes in phosphate-buffered saline (PBS). Coverslips were mounted onto slides with DAPI supplemented fluorescence mounting media, and observed by indirect immunofluorescence. Antibody binding was subjectively quantified as above. Following this initial screen, samples which gave positive staining were investigated further by testing after adsorption with pigeon acetone liver powder in order to remove any antibodies against non-CNS rat epitopes [21].

### Binding to live rat hippocampal neurons

Using established methods [20], neurons were prepared from P0 (newborn) rat pups. After 12 days in culture, coverslips with neuronal cultures were incubated with patient serum (1:100) and binding to the live neurons visualised by fixation and detection with Alexa Fluor 488 (green) as described previously. Any binding was scored subjectively in relation to a panel of healthy and positive controls.

## Results

### Clinical and CSF orexin data on patients

Clinical information and follow-up were only released after the orexin levels and antibody data were obtained. There were 9 males and 4 females (see Table 1). The time since symptom onset to serum/CSF sampling ranged from 7 to 41 months, median 14 months. Twelve patients had typical Type 1 narcolepsy (CSF orexin deficient) first presenting post-H1N1 vaccination. Patient #12, a 24 year old man, developed narcolepsy 18 months pre-vaccination but his cataplexy symptoms increased markedly following H1N1 vaccination.

All these subjects had the classical HLA DQB1*06:02 allele,

### Antibodies to known neuronal surface antigens

None of the CSFs or sera showed IgG antibodies binding to NMDAR, CASPR2, LGI1, dopamine receptors or GABA_A_ α3 receptor (Table 1).

### Antibodies to neuronal tissue

**Fig 1:**
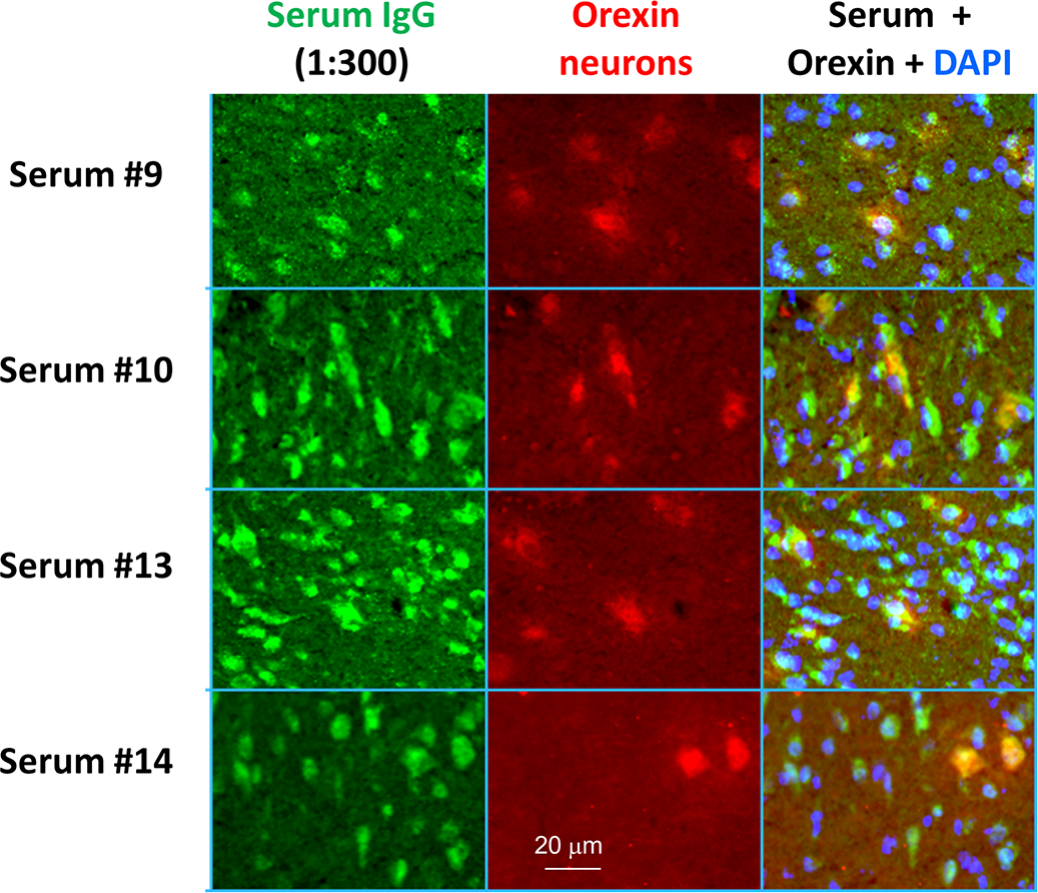
Binding of IgG from patients to orexin neurons on paraformaldehyde-fixed rat brain tissue sections. Co-localisation of serum IgG binding and orexin immunoreactivity was optimised using autoimmune disease and healthy control sera. In four marcolepsy patient sera, identified as reactive on the initial immunofluorescence screen, the patient serum IgG binding co-localised with commercial antibody to orexin, but there was also binding to other non-orexin neurons.

**Fig 2:**
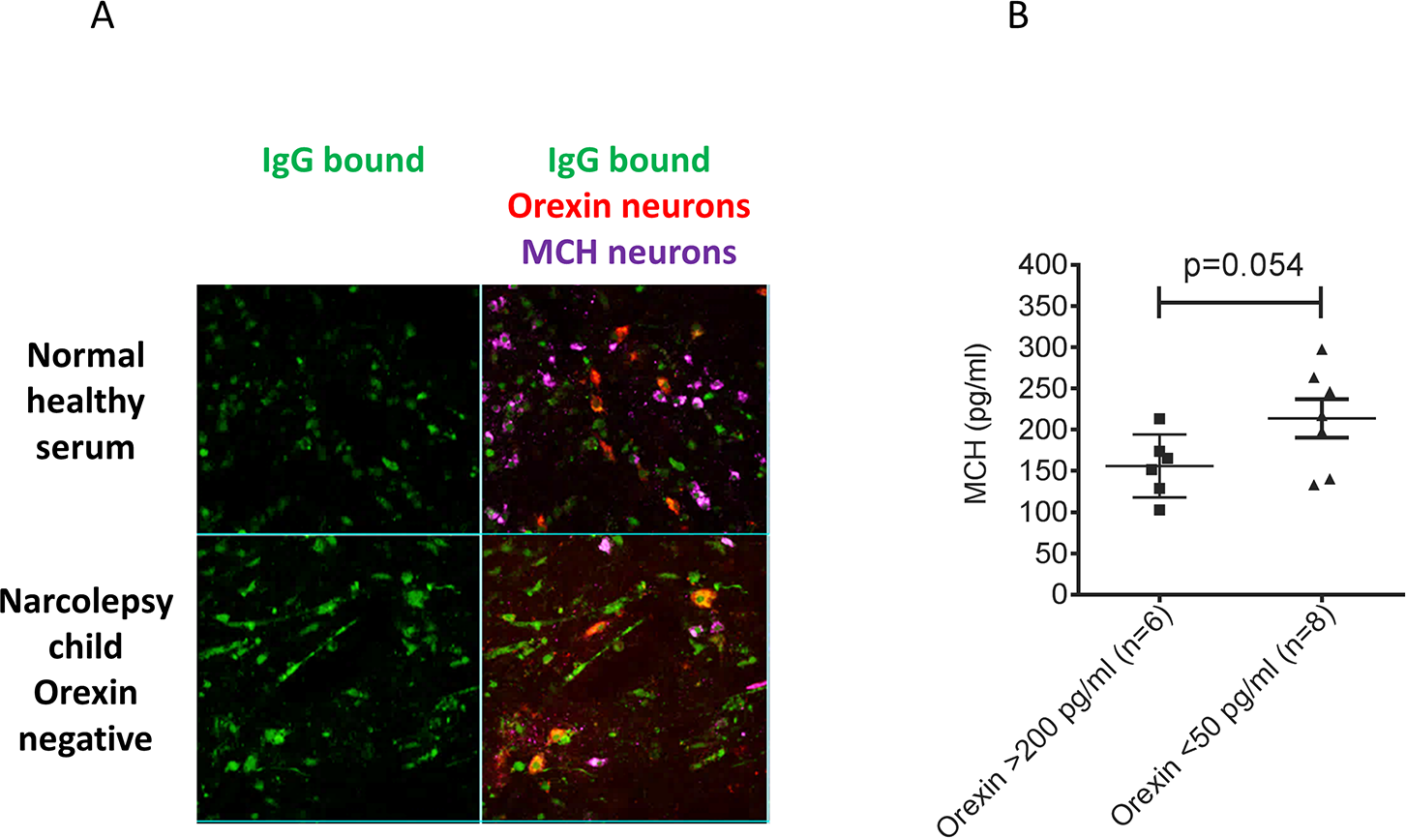
A. Binding of IgG to orexin and MCH neurons and CSF MCH levels in narcolepsy patients. Co-localisation of serum IgG binding (green), orexin immunoreactivity (red) and MCH immunorecativity (purple) showed co-localization of IgG binding with both orexin and MCH neurons. B. MCH measurements in 8 available CSFs (including one without serum available) from post-H1N1-AS03-vaccinated orexin-deficient narcolepsy children compared with CSFs from 6 orexin-normal children.

Antibody binding to the hypothalamus was found in four narcolepsy patient sera (Fig 1), and not in the healthy control sera (data not shown), but this binding was not specific for orexin neurons, since MCH-positive neurons were also stained (Fig 2A), and similar binding was detected in other brain regions including the cortex and hippocampus (data not shown). None of the narcolepsy CSFs showed detectable IgG binding to the rat brain sections.

### MCH levels in CSF

CSF MCH levels were only measured in eight patients, and were variable but with a clear trend towards higher values in the orexin negative narcolepsy CSFs compared with orexin normal non-narcolepsy paediatric CSFs (p = 0.054, Student’s unpaired t-test; Fig 2B, Table 1) or adult normal CSFs tested in parallel (data not shown).

### Antibodies binding to live hippocampal neurons

None of the Type 1 narcolepsy patients were positive for binding to live hippocampal neurons, cultured from new-born rat pups.

### Serum IgG levels pre and post H1N1-AS03 vaccination

**Fig 3:**
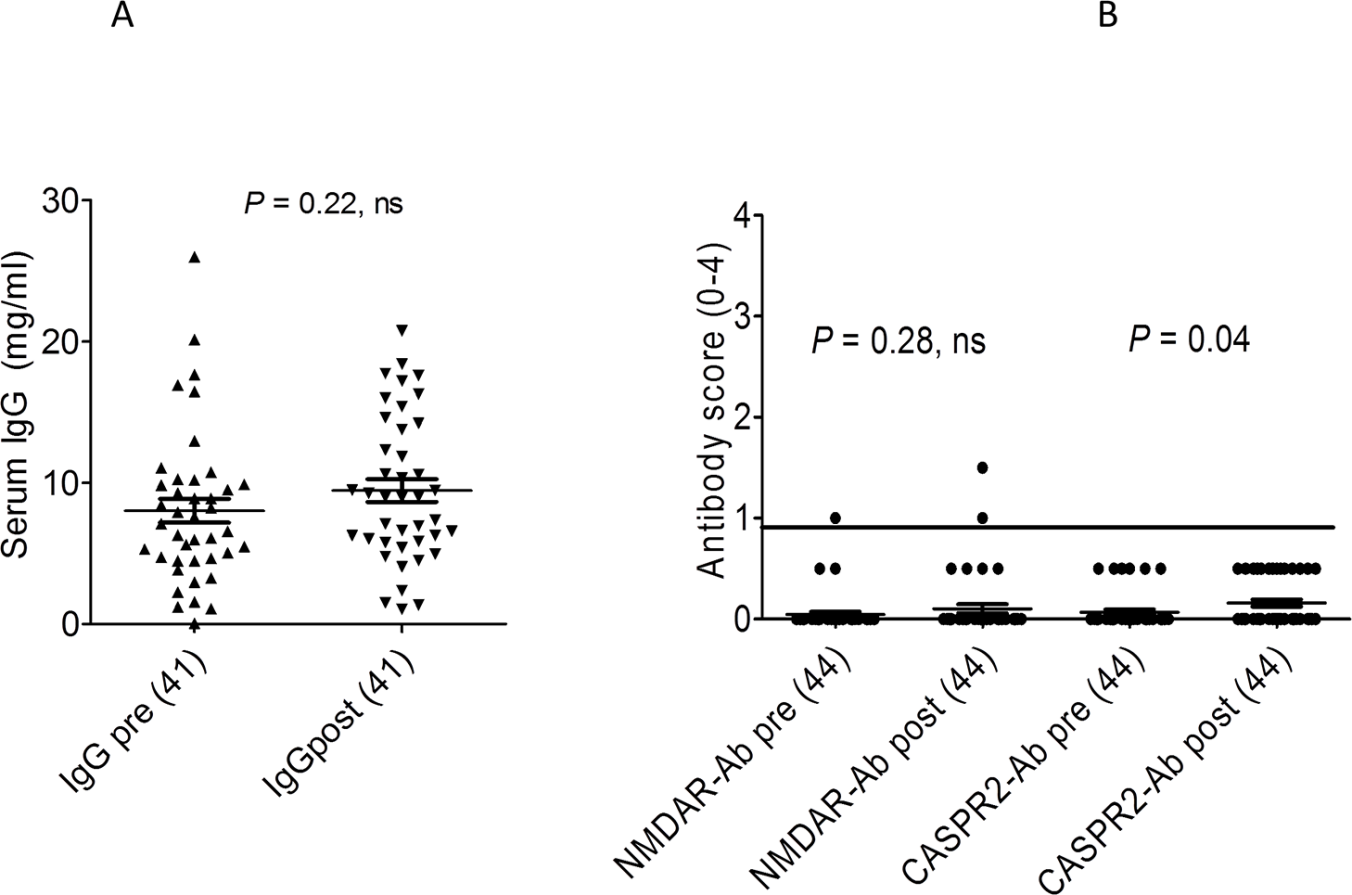
Results of total IgG and specific neuronal antibodies in available samples from pre and post-AS03-H1N1 vaccination study. The NMDAR antibody scores were not different between the pre- and post-vaccine patients although there were low positive results (scores 1 and 1.5) in two children post-vaccination. None of the CASPR2 antibodies reached the threshold score of 1 even though the average binding was slightly higher post-vaccine.

Total IgG levels were measured in sera from 42 healthy children [19] obtained pre and post-vaccination with H1N1-AS03 (age range 6 months to 12 years, mean age 4.8 months). Although the mean value post-vaccination was a little higher in the H1N1-AS03 vaccine recipients than prior to immunisation, this did not reach significance (p = 0.22; Fig 3A). To see if any antigen-specific antibodies had been induced by the vaccination, CASPR2 and NMDAR antibodies were measured. CASPR2 antibodies were insignificant (score <1) both pre- and post-vaccination, but NMDAR antibodies were weakly positive in one child pre- and post-vaccination, and one other child post vaccination only (Fig 3B) though these low values would not necessarily be considered of clinical relevance [22].

## Discussion

The role of autoantibodies in narcolepsy has been of interest for many years but no disease specific, potentially pathogenic, antibodies have been identified. The existence of a post-vaccination form of narcolepsy offered an opportunity to examine sera and CSFs for the presence of neuronal surface antibodies. Although no disease specific antibodies were found, four of 13 narcolepsy type 1 sera bound to orexin neurons, among other neurons, on brain tissue sections, suggesting some increase in neuronal autoimmunity. However, there was no evidence of specific antibodies in the AS03-H1N1 vaccination samples, and similarly there was no clear increase in autoimmunity or total IgG levels post-vaccination in the ASO3-H1N1 vaccination trial samples, although the numbers studied were relatively small and not many would have had the classical HLA DQB1*06:02 allele.

There have been many attempts [13] to identify antibody targets in narcolepsy, and others have identified hypothalamic hormone peptides [23] or orexin neuron-specific transcripts [24] that might prove to be narcolepsy-specific antibody targets, but immunostaining of rodent brain here only showed antibody binding to brain tissue in four of the post-vaccination narcolepsy children, and the binding was not orexin-cell specific. The complete absence of CSF reactivity with brain tissue sections or neuronal cultures was disappointing and could be interpreted as lack of evidence for any antibody-mediated pathogenesis in these patients, but since the immune response likely begins in the periphery the CSF levels could be very low and not easily detectable unless, or until, substantial intrathecal synthesis of the antibody occurs. Moreover, if the immune response rapidly destroyed the orexin/hypocretin neurons, antibodies may no longer be detectable either centrally or peripherally in samples such as these collected some months after the onset of narcolepsy symptoms.

The MCH neurons, which are normally interspersed between the orexin neurons and promote sleep, are not thought to be affected in narcolepsy [3]. A recent publication reported antibodies to a shared C terminal peptide of glutamic acid-isoleucine/a-melanocyte stimulating hormone (NE1/aMSH) which could alter MCH neuron functions in patients with narcolepsy[23], suggesting that secretion of the MCH peptide might be altered. Here, MCH levels in CSFs from orexin-deficient narcolepsy children showed a trend towards higher levels (p = 0.054) than in CSFs from orexin-normal children, although raised levels were not previously found in 14 patients with narcolepsy [25].

The study has obvious limitations. The number of subjects studied was small. Further candidate antigens or membrane proteins expressed in orexin cells could be explored further in the future. Unfixed tissue might provide a more suitable substrate than fixed brain sections to detect antibodies binding to highly conformational membrane proteins. To demonstrate cell-surface binding, use of live orexin-neurons would clearly be preferable to hippocampal neurons, but this would require further development.

Although the binding to brain tissue found in 4/13 patients may represent an increase in autoreactivity, this could occur as a secondary phenomenon following a T cell or other immune attack on the orexin neurons. Nevertheless, these studies did not exclude the possibility of a highly specific antibody, and as antibody-mediated diseases are usually responsive to immunotherapies [14] the topic remains an important area for future study.
